# Percutaneous Endoscopic Management for Oriental Cholangiohepatitis: A Case Report and a Brief Review of the Literature

**DOI:** 10.1155/2017/8575674

**Published:** 2017-08-27

**Authors:** Khalil Aloreidi, Prince Sethi, Terry Yeager, Muslim Atiq

**Affiliations:** ^1^Department of Internal Medicine, University of South Dakota Sanford School of Medicine, Sioux Falls, SD, USA; ^2^Department of Radiology, University of South Dakota Sanford School of Medicine, Sioux Falls, SD, USA; ^3^Department of Gastroenterology, University of South Dakota Sanford School of Medicine, Sioux Falls, SD, USA

## Abstract

Oriental cholangiohepatitis (OCH) is a disease characterized by intrabiliary pigment stone formation, resulting in recurrent bouts of cholangitis. OCH is found mostly in Southeast Asia but it is occasionally recognized in Western societies. OCH etiology is largely unknown. We report our experience with a patient who presented with acute cholecystitis. Following laparoscopic cholecystectomy, she developed acute cholangitis due to multiple biliary tree stones. She underwent ERCP to clear the stones from common bile duct. For the intrahepatic stones, she underwent novel hybrid percutaneous endoscopic technique. The procedure resulted in complete clearance of biliary tree stones and resolution of her symptoms. The aim of this case is to increase awareness of this disease when patients from endemic areas present with biliary stones.

## 1. Introduction

Oriental cholangiohepatitis (OCH) also called recurrent pyogenic cholangitis is a disease characterized by intrabiliary pigmented stones formation, resulting in biliary tree stricture and obstruction with recurrent bouts of cholangitis. OCH is found mostly in Southeast Asia (hence its name) but now occasionally recognized in Western societies with prevalence of less than 1% [1]. We report our experience with a patient who presented with extensive biliary tree stones and how we used a percutaneous endoscopic approach for stones extraction.

## 2. Case Report

**Figure 1:**
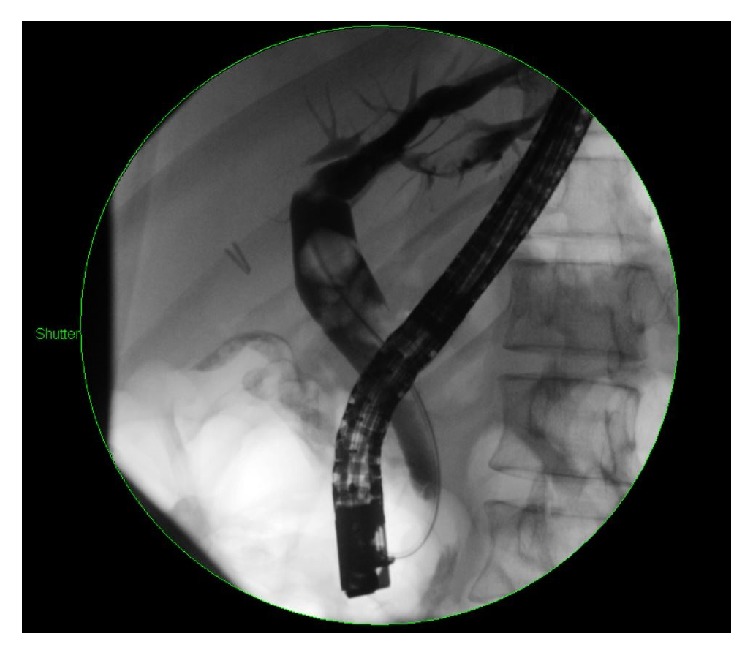
ERCP showing multiple CBD stones. Note that the right hepatic duct is not visualized due to obstruction.

**Figure 2:**
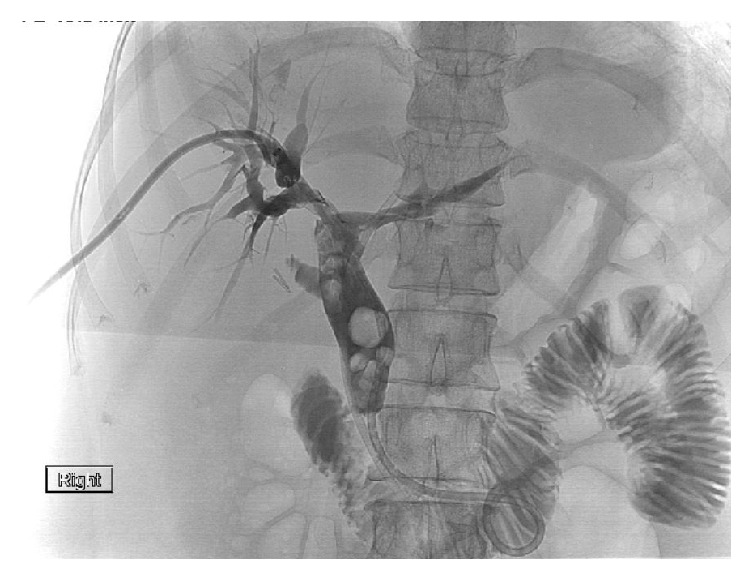
Percutaneous cholangiogram showing multiple stones in biliary tree.

**Figure 3:**
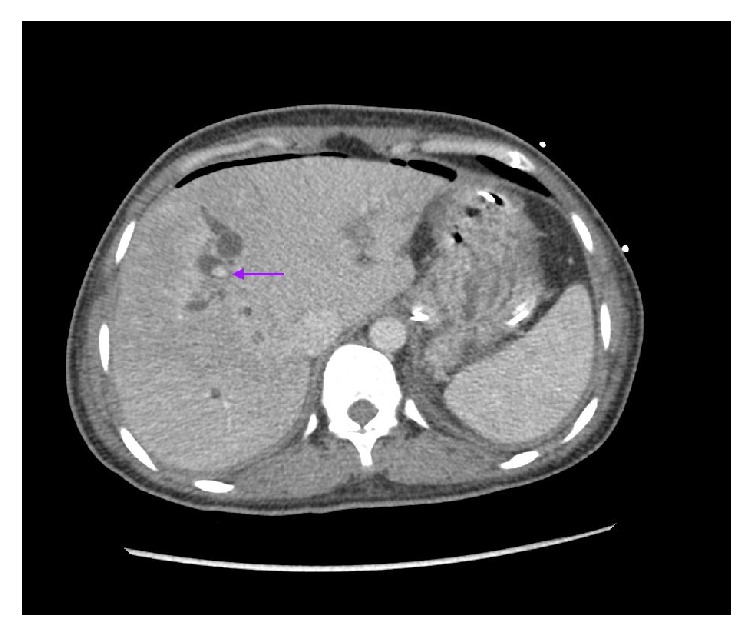
CT scan showing calcified stones within dilated intrahepatic ducts in the left lobe of the liver.

**Figure 4:**
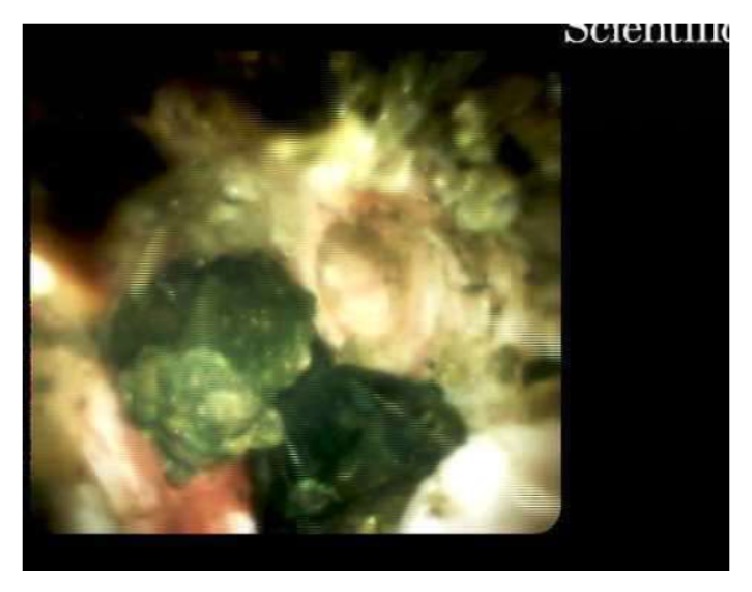
Cholangioscope image showing intrahepatic stone.

**Figure 5:**
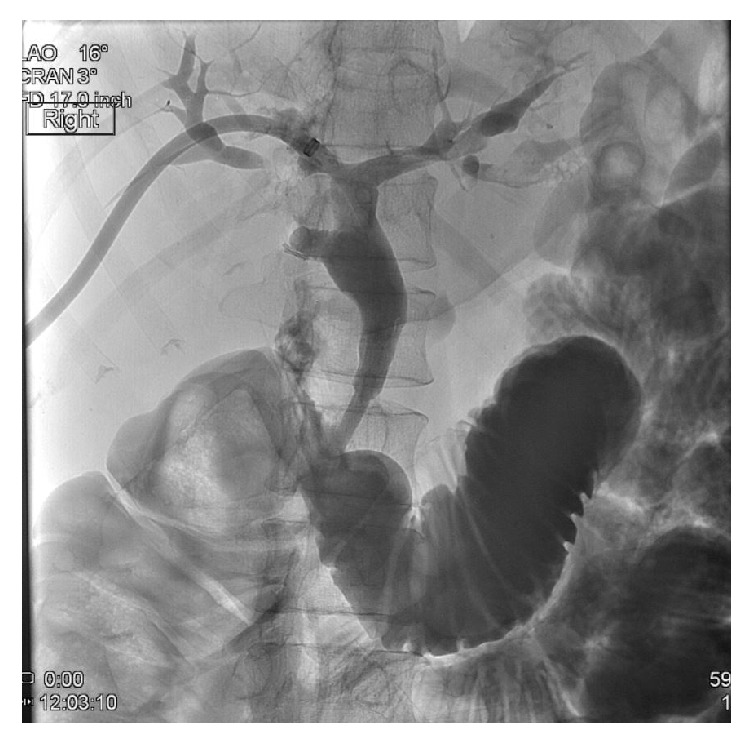
Percutaneous cholangiogram showing biliary tree after the procedure.

A 39-year-old Korean female presented with abdominal pain for 5 days. She was found to have acute cholecystitis after gallbladder ultrasound demonstrated cholelithiasis with wall inflammation. She underwent laparoscopic cholecystectomy with intraoperative cholangiogram which demonstrated choledocholithiasis. The following day, the patient developed fever and hypotension with elevated bilirubin level of 3.3 mg/dL. Her alkaline phosphatase was 174 U/L, alanine aminotransferase 215 U/L, and aspartate aminotransferase 126 U/L. She was started on IV antibiotics and had an urgent endoscopic retrograde cholangiopancreatography (ERCP) which demonstrated a common bile duct (CBD) stones with multiple intrahepatic stones as well (Figure 1). The biliary tree was swept with a balloon and pus came out from the duct. Subsequently, a temporary stent was placed in the CBD extending into the left biliary. Also, the right intrahepatic duct drain was placed by interventional radiology (Figure 2). An abdominal computer tomography (CT) scan with contrast showed stones within intrahepatic ducts on the left lobe of the liver (Figure 3). Given the ethnic background and the typical clinical picture, the patient was diagnosed as a case of oriental cholangiohepatitis with a plan for stone extraction through a combined percutaneous endoscopic approach. Later the drain and stent were removed and ERCP with sphincterotomy was done and all stones were cleared from CBD. From the percutaneous drain, a 16 French sheath was placed in the left lateral duct segment. Then cholangioscope was introduced through the sheath and electrohydraulic lithotripsy (EHL) was done under direct visualization (Figure 4) and stones were basketed from the left intrahepatic system. Similarly, the right anterior segment stones were disintegrated and extracted through the same technique. Cholangiogram showed no residual stones (Figure 5). The percutaneous drain was kept in place and a follow-up CT abdomen showed no intrahepatic stones. Later the drain was removed and the patient did well postoperatively.

## 3. Discussion

OCH is characterized by intractable nature and frequent recurrence requiring multiple operative interventions. In addition to frequent cholangitis and chronic sepsis, it is widely known that longstanding intrahepatic stones lead to intrahepatic cholangiocarcinoma which occurs in 5% of the cases [2]. The etiology of OCH is uncertain, although ethnic factors, bacterial infection, parasite infestation, and anomalies of the bile duct anatomy are implicated.

OCH usually affects the left hepatic duct, especially the left lateral segmental duct, in the early course of the disease for unknown reason [3], although stones may be present in the right and left hepatic lobes and the extrahepatic biliary tree. Given the difficult access to parts of biliary tree to which the disease is commonly distributed, the treatment usually complicated and needs multidisciplinary approach involving interventional radiology, interventional endoscopy, and surgery. Surgical resection of the affected liver segment has been reported to be effective [4], but sometimes surgery is not an option especially if the disease is not localized. Instead, the use of fluoroscopy combined with cholangioscope to directly visualize intrahepatic stones and duct strictures has been proved to be highly successful in stone clearance [5]. With this method the operator will be able to perform balloon dilatation of the biliary stricture and to use EHL to disintegrate the large stones or when the stones were impacted behind the strictures. However, the procedure has certain complications including liver laceration and intra-abdominal abscess due to biliary leakage.

In conclusion, percutaneous endoscopic approach is relatively safe alternative therapy for intrahepatic stones extraction. Long-term follow-up is required, because the overall recurrence rate for intrahepatic stones and/or cholangitis is high (63.2%) especially in patients with bile duct stricture. Also, complete clearance of intrahepatic stones is crucial as the incidence of cholangiocarcinoma is significantly higher in those with residual stones [5].
